# Comparative analysis of risk factors associated with degeneration of adjacent segments: zero-profile anchored spacer vs. anterior cervical plate and cage construct

**DOI:** 10.3389/fmed.2024.1375554

**Published:** 2024-06-03

**Authors:** Zhikai Wu, Wenhao Wang, Feng Zhou, Pan Xiang, Yangfeng Li, Huilin Yang, Genglei Chu

**Affiliations:** ^1^Department of Orthopaedic Surgery, The First Affiliated Hospital of Soochow University, Suzhou, Jiangsu, China; ^2^Orthopaedic Institute, Suzhou Medical College, Soochow University, Suzhou, China

**Keywords:** adjacent segment degeneration, anterior cervical discectomy and fusion, spine, cervical degenerative disc disease, zero-profile anchored spacer

## Abstract

**Objective:**

Anterior cervical discectomy and fusion (ACDF) is an established treatment for cervical degenerative disc disease, but cervical spine surgery may affect sagittal alignment parameters and induce adjacent segment degeneration (ASD). This study aimed to determine the risk factors for developing ASD following anterior cervical plate and cage (ACPC) compared with the use of zero-profile anchored spacer (ROI-C).

**Methods:**

A retrospective contrastive study included 105 patients who underwent ACPC or ROI-C between January 2014 and October 2019 at our treatment centre. There were 50 cases in the ROI-C group and 55 patients in the ACPC group. Clinical and radiological results and the incidence of ASD were assessed after surgery. All patients were further divided into the ASD and non-ASD groups for subgroup analysis.

**Results:**

At each follow-up time, there was no statistically significant in radiographic parameters between the two groups. The overall ASD rate was higher in the ACPC group than in the ROI-C group (65.5% vs. 44.0%, *p* = 0.027). The low preoperative Cobb angle, low preoperative segment angle (SA), and loss of Cobb (ΔCobb) were significantly correlated with ASD. However, clinical outcomes were not associated with ASD at any postoperative follow-up visit.

**Conclusion:**

Equally good therapeutic effects were achieved with both the ROI-C and ACPC. The occurrence of ASD was considerably higher in the ACPC group than in the ROI-C group. The preoperative Cobb angle, preoperative SA, and ΔCobb were the most associated with an increase in the risk of ASD.

## 1 Introduction

Cervical spondylosis is an age-related problem caused by degeneration or herniation of the intervertebral discs that compress the nerve roots or spinal cord (1). Anterior cervical decompression and fusion (ACDF) is an option commonly applied to cervical spondylosis caused by disc herniation or ossification of the posterior longitudinal ligament, which provides adequate access to the compression of the spinal cord, improves localised kyphotic deformity, and re-establishes sagittal alignment of the cervical spine (2). However, long-term arthrodesis of the spinal segments may lead to excessive biological stress at the unfused levels, and there is evidence that a proportion of 25–89% of patients will again have clinical symptoms in the adjacent segment that might require secondary surgery (3–5). The main manifestation of adjacent segment degeneration (ASD) is the reduction in height of the intervertebral space that may allow for narrowing of the intervertebral foramen, ossification of the posterior longitudinal ligament, reduced mobility of the fused segment, and reappearance of cervical spondylosis symptoms. Previous reported risk factors related to the age of the patient, cervical spine sagittal alignment range of motion, the location and number of fusion segments, and previous existing degenerative changes of the spine segments (6–8).

This research was carried out to evaluate the risk factors for ASD by analysing patients with anterior cervical arthrodesis treated with zero-profile anchored spacer (ROI-C) and anterior cervical plate and cage (ACPC). A retrospective study of 105 patients with cervical spondylopathy was performed with the comparison of long-term clinical follow-up and imaging data. The main purpose of this study was to investigate the long-term clinical effects of the ROI-C and ACPC in the treatment of cervical spondylosis as well as to explore radiologic results and risk factors associated with ASD.

## 2 Materials and methods

### 2.1 Ethics statement

This study is a retrospective, single-centre, open-label case series. The study was approved by the Medical Ethics Committee of The First Affiliated Hospital of Soochow University (ethical code 2020199). All participants provided their informed written consent to participate. All methods were performed in accordance with the Declaration of Helsinki.

### 2.2 Materials

In total, 105 patients who underwent ACDF for the treatment of degenerative cervical disease in our centre from January 2012 to October 2018 and had at least 6 years of follow-up were enrolled in the retrospective study. Patient inclusion criteria for this study were as follows: (1) the presence of cervical spondylosis or neurogenic cervical spondylosis due to cervical disc degeneration; (2) the failure of conservative treatment for at least 6 months. Exclusion criteria: (1) severe facet joint degeneration; (2) congenital cervical spinal stenosis; (3) cervical abnormalities; (4) severe unstable cervical spine with vertical displacement >2 mm or with angular displacement >2°; (5) posterior longitudinal ligament ossification; (6) cervical tumour or infection; and (7) a history of cervical spine surgery.

A total of 105 patients were included in this study; patients either received ROI-C (50 cases) or underwent ACPC (55 cases). Patients’ images and data were obtained from the Picture Achieving and Communication System or Electronic Medical Record Management System of The First Hospital of Soochow University, including imaging data, surgical procedure, demographic characteristics, Body Mass Index (BMI), blood loss, and hospital stay.

### 2.3 Surgical method

All surgical procedures were conducted by the same surgeon in our study. Surgeries were performed by conventional techniques as previously described by our orthopaedic centre. A classic Cloward and Robinson anterior technique and approach were performed. The symptomatic disc, osteophytes, and posterior longitudinal ligament were removed for extensive decompression of nerve roots and spinal cords. In order to expose the bony endplates for the prevention of possible cage subsidence, the cartilage endplates were abraded carefully. For the ROI-C group, a proper size of intervertebral fusion cages (ROI-C, LDR, Troyes, France) packed with bone induction and autologous cancellous bone was inserted into the disc place. Caspar’s intervertebral braces were loosened, and two anchoring chips were inserted into the lower and upper vertebral body for fixation whilst the fluoroscopy position was satisfactory. For the ACPC group, the PEEK-Cage (DePuy Co.) filled with bone induction and autologous cancellous bone was placed into the intervertebral space along with the dynamic compression titanium plate. All processes above are performed under C-arm guidance.

### 2.4 Clinical evaluation

Patients were evaluated clinically before the surgery, 3 months postsurgery, and at the final follow-up visit at more than 60 months. The Japanese Orthopaedic Association (JOA) scores reflected the clinical symptoms in terms of limb movement, sensation, and bladder function. Neck Disability Index (NDI) scores were used to assess the influence of cervical degenerative disease before and after surgery. Using a Visual Analogue Scale (VAS), the impact of the patient’s neck discomfort was determined. The score progressed from smaller to larger with symptoms progressing from mild to severe.

### 2.5 Radiological evaluation

**Figure 1:**
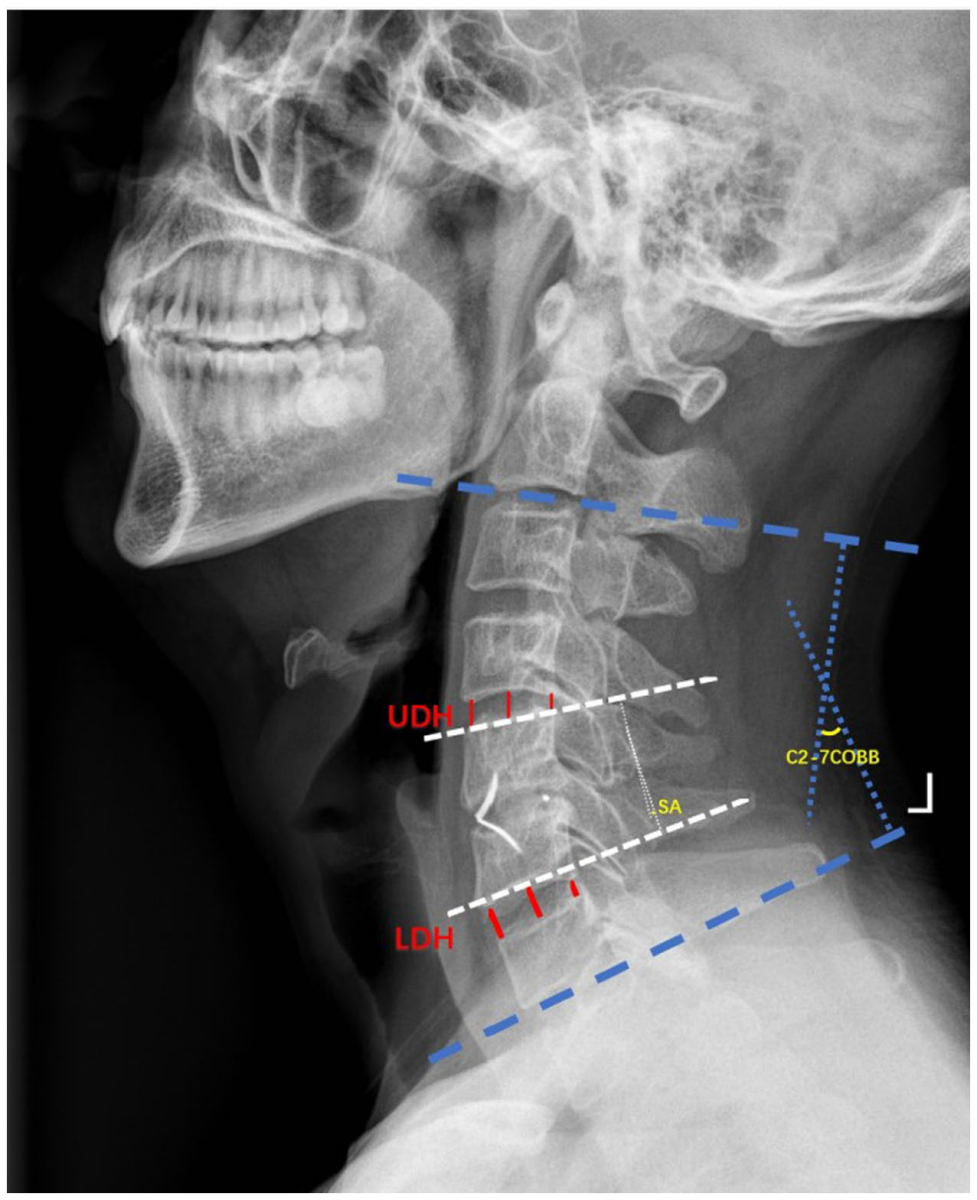
Illustrations of radiographic parameters. Cervical Cobb angle, T1 slope; segment angle (SA); upper disc height (UDH); lower disc height (LDH).

All images were downloaded and imported into Digimizer software (MedCalc Software Ltd., Version 4.3.5.0) for radiological assessment of cervical spine angulation. The radiologic outcomes included Cobb angle, segment angle (SA), C2-C7 range of movement (C2-C7 ROM), lower segmental range of movement (LSROM) and upper segmental range of movement (USROM), lower segment disc height (LDH), and upper segment disc height (UDH). The cervical Cobb angle was measured in the standard lateral radiograph by drawing a straight line from the inferior endplate of the C2 vertebral body and the superior endplate of the C7 vertebral body. The SA was measured using the same method as for the Cobb angle as measured by drawing a straight line from the lower endplate of the fusion levels and the upper endplate of the fusion levels. The LSROM or USROM was measured by analyse the angle between the upper endplate and lower endplate of the lower or upper adjacent segment disc in the dynamic X-ray. The LDH or UDH was measured by analyse the distance between the centre of the superior endplate of the lower vertebral body and the inferior endplate of the upper vertebral body (Figure 1).

### 2.6 Assessing adjacent segment degeneration

Adjacent segment degeneration (ASD) was evaluated by standard frontal and lateral radiographs and magnetic resonance imaging (MRI). The Kellgren X-ray cervical vertebral degeneration system was used to evaluate the degenerative changes that include a > 25% reduction in disc height, new anterior or posterior osteophyte formation, calcification of the anterior longitudinal ligament (>3 mm), and endplate sclerosis (9, 10). The new disc herniation showed by T2-weighted MRI was also used to define the appearance of ASD.

The cases whose plain film radiographs and MRI appeared to show adjacent segment degeneration were further included in the ASD group, and the remaining patients were included in the non-adjacent segment degeneration (non-ASD group). Afterward, the risk factors associated with the incidence of ASD were assessed in the ASD and non-ASD groups. The analysis factors include age, surgical methods, preoperative Cobb angle, and change in Cobb (ΔCobb = postoperative Cobb-preoperative Cobb).

### 2.7 Statistical analysis

Data processing was performed using SPSS 22.0 software (IBM Corporation, USA). *T*-tests were used for comparisons, whereas *chi*-squared tests were used to analyse data on categorical variables. Logistic regression analysis was performed for risk factors associated with ASD that exhibited significance. Differences were considered statistically significant when *p* < 0.05.

## 3 Results

### 3.1 Demographics and perioperative outcomes

A total number of 105 patients were considered eligible for enrolment in this study, including 49 men and 56 women, who underwent zero-profile anchored spacer (ROI-C) or anterior cervical plate and cage (ACPC). Surgery was completed successfully without neural damage, implant displacement, prosthesis collapse, hematoma, oesophageal injury, infection, or other complications.

In the ROI-C group, the follow-up time and mean age were 72.02 ± 16.82 months and 52.88 ± 11.41 years old, respectively. In the ACPC group, the follow-up time and mean age were 78.75 ± 9.96 months and 50.56 ± 10.06 years old, respectively. There were no statistically significant differences in patients’ number, age, sex, BMI, and number of operated levels (*p* > 0.05). Compared with the ACPC group, the ROI-C group was associated with a shorter operation time (139.72 ± 76.99 min for ROI-C vs. 170.22 ± 68.15 min for ACPC; *p*<0.05) and lower estimated blood loss (69.61 ± 58.59 min for ROI-C vs. 94.36 ± 49.77 min for ACPC; *p*<0.05). The length of hospital stay was slightly longer in the ACPC group than the ROI-C group, but there was no statistically significant difference between the two groups (*p* > 0.05). Patient demographics are summarised in Table 1.

**Table 1:** Demographic data and perioperative parameters of the two groups.

Variables	ROI-C	ACPC	*p*-value
Cases (*n*)	50	55	
Age (years)	52.88 ± 11.41	50.56 ± 10.06	0.269
Sex (male/female)	25/24	24/32	0.391
BMI (kg/m^2^)	24.09 ± 3.20	23.88 ± 2.66	0.713
Number of fused levels (n)			
1	29	31	0.795
≥2	21	24	
Operation time (min)	139.72 ± 76.99	170.22 ± 68.15	0.033^*^
Blood loss (ml)	69.61 ± 58.59	94.36 ± 49.77	0.021^*^
Hospital stay (days)	12.54 ± 3.53	13.60 ± 3.18	0.107

BMI, body mass index.

^*^Statistically significant difference (*p* < 0.05).

### 3.2 Clinical outcomes

**Figure 2:**
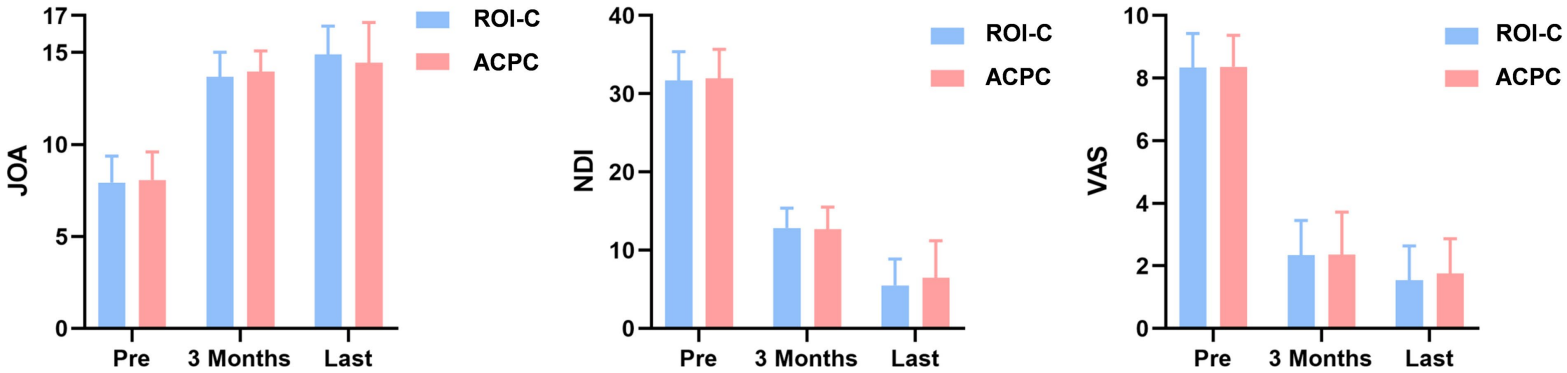
JOA, NDI, and VAS scores between the ROI-C and ACPC groups.

Compared with the baseline data, both groups showed significant improvement in VAS, NDI, and JOA scores at 3 months postoperatively and the final follow-up (*p* < 0.05). No statistically significant difference was found in VAS, JOA, and NDI scores between the two groups during the follow-up time (*p* > 0.05, Figure 2).

### 3.3 Cobb angle and overall range of movement (ROM) activity

The Cobb angle, SA value, and the overall activity or range of movement (ROM) between the ROI-C and ACPC groups showed no significant difference at each follow-up time point (*p* > 0.05). In the final follow-up, the Cobb angle and C2-C7 ROM in the ROI-C group were more than in the ACPC group, but with no statistically significant difference (*p* > 0.05). The SA values for both groups increased significantly at each time point postoperatively (*p* < 0.05), with no significant differences observed between the two groups at each time point, indicating the restoration of disc height. However, for both groups, reductions in cervical Cobb angle and SA value were found at the final follow-up compared with the postoperative values. The Cobb angle, SA, and C2-7 ROM values for both groups were well maintained postoperatively at the last follow-up. The detailed data are shown in Table 2.

**Table 2:** Cobb angles, SA, and overall activity of the ROI-C and ACPC groups.

Variables	ROI-C (50 cases)	ACPC (55 cases)	*p*-value
Cobb angle (°)			
Preoperatively	13.41 ± 10.24	15.60 ± 10.43	0.296
3 months	17.80 ± 9.58^#^	17.18 ± 9.40	0.782
Final FU	16.15 ± 10.87	15.98 ± 10.59	0.944
SA (°)			
Preoperatively	5.28 ± 6.08	5.97 ± 6.89	0.688
3 months	10.03 ± 5.44^#^	10.11 ± 6.68^#^	0.953
Final FU	7.61 ± 5.61^#^	9.63 ± 6.35^#^	0.103
C2-C7 ROM (°)			
Preoperatively	42.12 ± 4.91	43.62 ± 6.42	0.201
3 months	36.44 ± 5.20	35.09 ± 6.38	0.269
Final FU	39.34 ± 6.08	37.93 ± 5.70	0.220

SA, segment angle; C2-C7 ROM, C2-C7 range of movement.

^*^Statistically significant difference (*p* < 0.05).

^#^Significance compared with preoperative (*p* < 0.05).

### 3.4 Adjacent segment intervertebral disc mobility and height

There was no significant difference in the preoperative upper and lower adjacent intervertebral in both groups. The intervertebral height showed no significant difference at the last follow-up when compared with preoperative status. At the final follow-up, reduction was found in the upper and lower segmental mobility compared with preoperative mobility in both groups, but there was no statistically significant difference (*p* > 0.05) (Table 3).

**Table 3:** Adjacent segment height and range of movement between the two groups.

Variables	ROI-C (50 cases)	ACPC (55 cases)	*P*-value
USROM (°)			
Preoperatively	9.62 ± 1.57	10.19 ± 1.82	0.103
3 months	7.85 ± 1.35	7.48 ± 1.24	0.160
Final FU	8.01 ± 1.64	7.52 ± 1.96	0.196
LSROM (°)			
Preoperatively	9.42 ± 1.74	9.65 ± 1.49	0.489
3 months	7.57 ± 1.15	7.13 ± 1.83	0.197
Final FU	8.28 ± 1.44	7.92 ± 1.20	0.165
UDH (mm)			
Preoperatively	12.59 ± 1.24	12.17 ± 1.35	0.101
3 months	12.11 ± 1.69	12.23 ± 1.94	0.737
Final FU	11.85 ± 1.12	11.80 ± 1.83	0.868
LDH (mm)			
Preoperatively	13.66 ± 1.52	13.79 ± 1.60	0.671
3 months	13.11 ± 1.17	12.91 ± 1.46	0.443
Final FU	14.14 ± 1.27	13.75 ± 1.92	0.227

USROM, upper segmental range of movement; LSROM, lower segmental range of movement; LDH, lower segment disc height; UDH, upper segment disc height.

^*^Statistically significant difference (*p* < 0.05).

### 3.5 Adjacent segment degeneration

**Figure 3:**
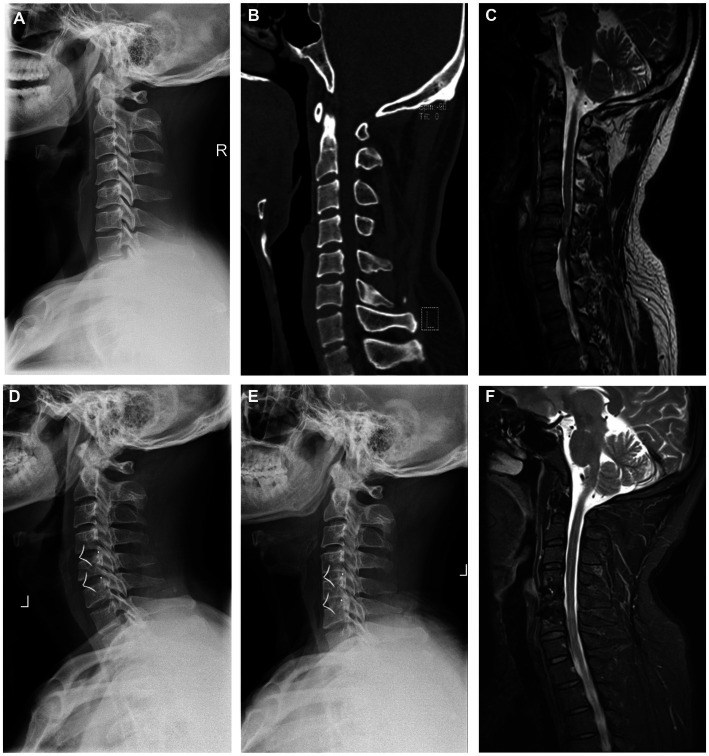
Images of a 49-year-old male patient at 88 months after anterior cervical zero-profile anchored spacer (ROI-C) surgery. Preoperative lateral cervical DR **(A)**, CT **(B)**, and MRI **(C)** indicate C4–C6 disc herniation with obviously spinal cord compressing. **(D–F)** Postoperative radiograph showed a good position of the internal fixation and corrected sagittal alignment at the final follow-up.

**Figure 4:**
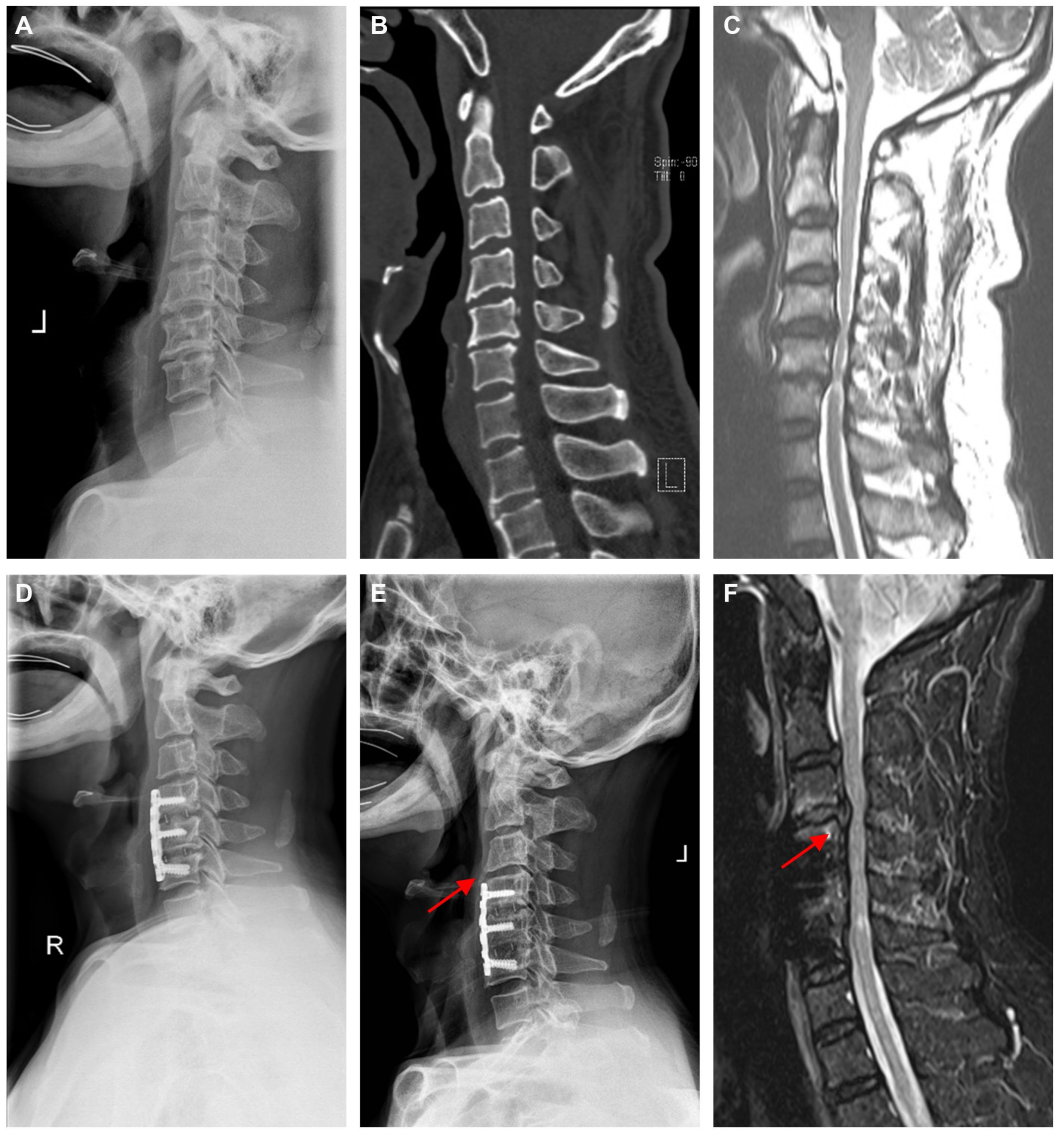
Images of a 66-year-old male patient who 79 months after anterior cervical plate and cage (ACPC) surgery. **(A–C)** Preoperative imaging revealed spinal cord compression at C4-C6, which was consistent with the symptoms. **(D)** Postoperative X-ray of 3 months indicates a relatively normal sequence of vertebrae. **(E)** At the last follow-up, the adjacent segment had decreased disc height and osteophyte formation anterior to the intervertebral space. **(F)** MRI suggested disc herniation over the operated segment, and clearly, compression of the spinal cord was observed at the last follow-up, as marked by the red arrow.

MRI-T2-weighted imaging and X-ray were used to evaluate ASD. The progression of ASD was judged when an increase in MRI or X-ray parameters was detected between two time points. Patients with the radiographic parameters that showed degeneration were divided into the ASD group at the last follow-up. Illustrative cases are shown in Figures 3, 4. There were 58 patients in the ASD group (incidence: 55.24%). We compared the ASD group with the non-ASD group and found that the sex, number of levels fused, BMI, and ΔSA showed no significant difference (*p* > 0.05). The average age of the ASD group (52.97 ± 10.54 years) was older than that of the NASD group (50.09 ± 10.90 years) but with no statistically significant difference (*p* > 0.05, Table 4). In the ASD group, the preoperative Cobb angle and SA were less than in the non-ASD group (*p* < 0.05). In addition, the loss of postoperative Cobb (ΔCobb) was significantly more in the ASD group than in the non-ASD group (Table 5).

**Table 4:** Baseline data comparison of the ASD and non-ASD groups.

Variables	ASD (58 cases)	Non-ASD (47 cases)	*p*-value
Sex (male/female)	28/30	21/26	0.681
Age (years)	52.97 ± 10.54	50.09 ± 10.9	0.173
BMI (kg/m^2^)	24.01 ± 2.83	23.94 ± 3.05	0.903
Surgical methods			
ROI-C	22 (44.0%)	28 (56.0%)	0.027^*^
ACPC	36 (65.5%)	19 (34.5%)	
Number of operated levels			0.962
1	33	27	
≥2	25	20	

BMI, body mass index.

^*^Statistically significant difference (*p* < 0.05).

**Table 5:** Preoperative radiographic parameters of the ASD and non-ASD groups.

Variables	ASD (58 cases)	Non-ASD (47 cases)	*p*-value
Cobb angle (°)	7.7 ± 2.93	15.1 ± 3.06	<0.001^*^
SA (°)	5.7 ± 2.54	7.2 ± 2.05	0.001^*^
C2-C7 ROM	43.3 ± 5.54	45.0 ± 6.22	0.149
UDH (mm)	11.9 ± 2.79	12.5 ± 2.66	0.266
USROM (°)	9.9 ± 2.54	10.8 ± 3.11	0.115
LDH (mm)	12.8 ± 1.77	13.4 ± 2.76	0.181
LSROM (°)	7.7 ± 1.44	8.0 ± 1.48	0.320
ΔSA	1.36 ± 3.77	1.52 ± 4.04	0.815
ΔCobb	2.58 ± 8.05	−0.03 ± 8.11	0.038^*^

SA, segment angle; C2-C7 ROM, C2-C7 range of movement; USROM, upper segmental range of movement; LSROM, lower segmental range of movement; LDH, lower segment disc height; UDH, upper segment disc height; ΔCobb, postoperative Cobb-preoperative Cobb; ΔSA, postoperative SA-preoperative SA.

^*^Statistically significant difference (*p* < 0.05).

### 3.6 Risk factors for adjacent segment degeneration

We discovered the following factors to be related to the increase in the risk of ASD after univariate analysis: age, preoperative Cobb angle, preoperative SA, ΔCobb, and the use of ACPC. These risk factors were examined by using multiple logistic regression. The results showed that the occurrence of ASD was significantly associated with the older age and the use of ACPC. Moreover, cervical C2-C7 Cobb loss of more than 5.5° is an important risk factor for ASD after ACDF (*p* < 0.05, OR = 3.547) (Table 6).

**Table 6:** Multifactorial analysis of ASD risk factors.

Variables	Odds ratio (95% CI)	*p*-value
Surgical methods (ROI-C vs. ACPC)	0.376	0.022
Age (<50 vs. ≥50)	1.386	0.440
Preoperative Cobb	13.245	<0.001
ΔCobb (<5.5°vs. 5.5°)	3.547	0.014

ΔCobb postoperative Cobb-preoperative Cobb.

## 4 Discussion

In our current study, the patient-reported outcomes showed that the long-term clinical effect of ROI-C compared with the use of ACPC achieved equal clinical outcomes that included 58 patients with adjacent segment degeneration (ASD). The postoperative neurological symptoms for both surgical groups were alleviated because of the sufficient decompression of the nerve root and spinal cord, and there was no significant differences were observed in either the JOA, NDI, or VAS scores between the two groups at different time points during the postoperative follow-up.

Although the advent of ACDF has brought great benefits to patients suffering from cervical spondylosis, it is important to bear in mind that ACDF is also inevitably associated with complications, such as subsequent instability, loss of physical activity, and ASD. The major pathological changes of ASD include ossification, ligament hypertrophy, disc herniation, vertebral slipping, disc space narrowing, development of cervical osteophyte around the vertebral body, and cervical spondylosis on MRI-T2 or X-ray images (11, 12). These degeneration changes of adjacent and fused segments may lead to cervical stenosis, which causes neurological symptoms due to the spinal cord injury (13, 14). ASD is related to the altered original mechanical behaviour and has a direct influence on the long-term clinical manifestation of patients who underwent ACDF surgery (15).

ACDF surgery may change the biomechanical environment of the spine caused by fusion and fixation, resulting in stress concentration of the adjacent vertebrae. Some studies have shown that ACPC is characterised by long-term maintenance of sagittal stability of the cervical spine, but the incidence of ASD cannot be ignored (16, 17). Biomechanical research has shown that plates placed in front of the vertebral body increase the stress on the adjacent vertebral body, whilst the ROI-C is designed to reduce the increased stress by fitting within the intervertebral space, thereby reducing degeneration of adjacent segments (18, 19). In this study, the final postoperative follow-up in both the ROI-C and ACPC groups did not reveal a significant difference in the sagittal alignment changes of the cervical spine. So far, there is still no consensus on whether ASD is a natural progression of degeneration in the cervical spine or a change in biomechanics after ACDF surgery (13, 20, 21). Several research findings suggest that age and ASD are closely related. Several studies have shown a higher incidence of postoperative ASD in the older (>50 years old) group than in the younger (<50 years old) group (16). However, some scholars hold a different opinion that being younger than 50 years significantly contributes to ASD because of a longer survival time (10, 22). According to the follow-up of at least 6 years, the mean age of the ASD group was older than that of the non-ASD group, but no significant difference was found between the two groups. The relationship between age and ASD is complex and might require more data on long-term clinical outcomes.

In previous studies, ASD was observed by plain film or CT scans, but plain film or CT scans cannot directly show the spinal cord compression and posterior margin (13, 23). In this study, MRI was used to determine spinal cord compression and intervertebral disc degeneration and to evaluate the grade of ASD. The MRI data were analysed from various aspects, including posterior and anterior compression, the spinal cord sagittal diameter, and the loss of disc height. The combination of X-ray, CT, and MRI in this study was demonstrated to be a good method to observe ASD, showing that the occurrence of ASD using the ACPC (65.5%) was significantly higher than that for the ROI-C group (44.0%) when the treatment involved single-level or multiple-level surgeries.

Physiological cervical lordosis can protect the spinal cord and maintain spinal stability. The incidence of ASD is thought to be directly associated with the decrease in cervical curvature (24, 25). Some researchers showed that there was an association between postoperative kyphosis and the instability of the cervical spine; meanwhile, postoperative kyphosis is also associated with ASD so that it is essential to reconstruct and maintain cervical lordosis (26, 27). After ACDF, when there is excessive loss of cervical lordosis, the stress on adjacent segments increases in the orthostatic position, or during flexion and extension activities, leading to accelerated disc degeneration in adjacent segments. Some scholars have ascertained that maintaining Cobb stability will reduce the motion of adjacent segments and the onset of ASD is also reduced (4, 28). In this study, we constructed a retrospective study to explore cervical lordosis pre- and postoperation and found that ROI-C and ACPC surgery both maintained overall lordosis and segment angle. However, the preoperative cervical lordosis of the ASD group is less than that non-ASD group. In addition, the loss of postoperative Cobb was statistically more significant in the ASD group than in the non-ASD group. The analysis of multivariate logistic regression also suggested that ASD is more likely to occur if Cobb loss exceeds 5.5° after the surgery. Therefore, postoperative cervical bracing and long-term cervical muscle exercise might be good strategies to maintain postoperative cervical stability, which will also reduce the risk of ASD.

The underlying data in both groups found that the procedure time of ROI-C was significantly lower than ACPC, which could be attributed to the need to adjust the position of the screws and plates in the ACPC group, in addition to the extra time required to grind down or remove the anterior vertebral bone to flatten the anterior cervical plates (29). This not only increases the number of fluoroscopic views but also increases the possibility of increasing vertebral bleeding (30). Bucci et al. (18) showed that ROI-C has stable biomechanical properties, and may even have fewer complications than ACPC, which can reduce the patient’s postoperative dysphagia symptoms. So far, the relationship between ASD and clinical outcomes is still debatable (31). Previous studies have reported that there was no significant difference in clinical outcomes between patients with and without ASD. The clinical outcomes in our study, based on JOA, NDI, or VAS scores, reveal that both the groups improved after surgery. We can speculate that ASD after ACDF is not associated with clinical outcomes.

There were several limitations regarding this study. First, this study was a retrospective study and lack of randomisation between procedures. Second, this research did not include the intervertebral fusion rate and sagging of the cage as a risk factor for ASD. Finally, larger numbers of patients and longer follow-up time remain necessary to determine its impact on clinical outcomes and to evaluate ASD of ROI-C and ACPC.

## 5 Conclusion

Zero-profile anchored spacer (ROI-C) and anterior cervical plate and cage (ACPC) for the cervical degenerative disease have achieved comparable clinical outcomes in our study. Nevertheless, our study found that the occurrence of ASD was considerably higher in the ACPC group than the ROI-C group. We further identified that ASD was significantly associated with the use of ACPC, low preoperative Cobb angle, and Cobb loss of more than 5.5° is an important risk factor for ASD after ACDF.

## Data availability statement

The original contributions presented in the study are included in the article/supplementary material, further inquiries can be directed to the corresponding author.

## Ethics statement

The studies involving humans were approved by the First Affiliated Hospital of Soochow University (ethical code 2020199). The studies were conducted in accordance with the local legislation and institutional requirements. The participants provided their written informed consent to participate in this study.

## Author contributions

ZW: Writing – original draft, Data curation, Conceptualization. WW: Writing – original draft, Project administration, Methodology, Data curation. FZ: Writing –review & editing, Software, Project administration, Investigation, Formal analysis. PX: Writing – original draft, Investigation, Formal Analysis. YaL: Writing – review & editing, Validation, Supervision, Project administration. HYa: Writing – review & editing, Supervision, Resources, Formal analysis, Conceptualization. GC: Writing – review & editing, Writing – original draft, Visualization, Validation, Supervision, Software, Resources, Project administration, Methodology, Investigation, Funding acquisition, Formal analysis, Data curation, Conceptualization.
